# A Validated Mathematical Model of the Cytokine Release Syndrome in Severe COVID-19

**DOI:** 10.3389/fmolb.2021.639423

**Published:** 2021-07-20

**Authors:** Ruy Freitas Reis, Alexandre Bittencourt Pigozzo, Carla Rezende Barbosa Bonin, Barbara de Melo Quintela, Lara Turetta Pompei, Ana Carolina Vieira, Larissa de Lima e Silva, Maicom Peters Xavier, Rodrigo Weber dos Santos, Marcelo Lobosco

**Affiliations:** ^1^ Institute of Exact Sciences, Department of Computing, Federal University of Juiz de Fora, Juiz de Fora, Brazil; ^2^ FISIOCOMP - Laboratory of Computational Fisiology and High-Performance Computing, Federal University of Juiz de Fora, Juiz de Fora, Brazil; ^3^ Computer Science Department, Federal University of São João Del-Rei, São João Del-Rei, Brazil; ^4^ Institute of Education, Science and Technology of Southeast of Minas Gerais - Cataguases Advanced Campus, Cataguases, Brazil; ^5^ GET-EngComp, Grupo de Educação Tutorial Engenharia Computacional, Federal University of Juiz de Fora, Juiz de Fora, Brazil; ^6^ Graduate Program on Computational Modeling, Federal University of Juiz de Fora, Juiz de Fora, Brazil

**Keywords:** computational immunology, SARS-CoV-2, COVID-19, mathematical modelling, sensitivity analysis, citokine storm

## Abstract

By June 2021, a new contagious disease, the Coronavirus disease 2019 (COVID-19), has infected more than 172 million people worldwide, causing more than 3.7 million deaths. Many aspects related to the interactions of the disease’s causative agent, SAR2-CoV-2, and the immune response are not well understood: the multiscale interactions among the various components of the human immune system and the pathogen are very complex. Mathematical and computational tools can help researchers to answer these open questions about the disease. In this work, we present a system of fifteen ordinary differential equations that models the immune response to SARS-CoV-2. The model is used to investigate the hypothesis that the SARS-CoV-2 infects immune cells and, for this reason, induces high-level productions of inflammatory cytokines. Simulation results support this hypothesis and further explain why survivors have lower levels of cytokines levels than non-survivors.

## 1 Introduction

By the beginning of June 2021, the number of confirmed deaths caused by the novel coronavirus pneumonia, COVID-19, surpassed 3.7 millions, while more than 172 millions worldwide were infected. The causative virus was first identified as SARS-CoV-2, also referred to as HCoV-19. It has been shown that SARS-CoV-2 infects the alveolar epithelial cells, mainly type 2 alveolar epithelial cells (AEC2), via the angiotensin-converting enzyme receptor 2 (ACE2) (Catanzaro et al., 2020; Hoffmann et al., 2020; Shang et al., 2020). The ensuing destruction of the epithelial cells and the increase in cell permeability lead to the release of the virus. During the fight against the virus, the innate immune system cells release a large number of extracellular molecular regulators, like cytokines and chemokines, that will induce the adaptive response to recruit more cells from the innate system (Coperchini et al., 2020; Prompetchara et al., 2020). In most individuals, recruited cells (mainly CD8^+^ T cells) are sufficient to clear the infection. However, in some patients, a dysfunctional immune response occurs, which triggers a “cytokine storm” that mediates widespread inflammation and damage (Tay et al., 2020), mainly in the lung.

The Cytokine Release Syndrome (CRS) or cytokine storm has been associated with a wide variety of infectious and non-infectious diseases for the past decades, including influenza and SARS-CoV (Tisoncik et al., 2012; Shimabukuro-Vornhagen et al., 2018). Nevertheless, the exact signalling pathways that lead to the CRS are yet to be determined (Lukan, 2020). Several mechanisms have been proposed to explain the CRS and the differences between survivors and non-survivors concerning viral dynamics and the immune response to SARS-CoV-2. One hypothesis is that SARS-CoV-2 infects macrophages, CD4^+^ T cells, and CD8^+^ T cells in addition to alveolar epithelial cells (Davanzo et al., 2020; Grant et al., 2021), thus causing the production of several pro-inflammatory cytokines (mainly IL-6) and also impairments in the immune response mediated by macrophages and T cells. The infection of CD8^+^ T cells, for example, prevents those cells from killing other infected cells and induces high-level production of some inflammatory cytokines, including IL-6 (Li H. et al., 2020; Blanco-Melo et al., 2020; Chen et al., 2020).

In this work, the hypothesis above is tested by a mathematical model of the immune response to SARS-CoV-2. The model developed is an extension of a prior model of the adaptive immune response, which was validated using experimental data obtained from vaccination against yellow fever (Bonin et al., 2019). The original model considers the main cells and molecules present in the immune response, such as, for example, antigen-presenting cells, CD4^+^ and CD8^+^ T cells, B cells, and IgM and IgG antibodies. We further extended the model in this work, including pro-inflammatory cytokines and infected immune system cells. Also, the model is validated with experimental data of the viremia, antibodies (IgM and IgG), and cytokines obtained from patients with COVID-19 (Long et al., 2020; To et al., 2020; Zhou et al., 2020). Moreover, a sensitivity analysis (SA) revealed important characteristics of the immune response to SARS-CoV-2.

## 2 Related Works

A large number of works use mathematical and computational tools to model the Human Immune System (HIS) using distinct techniques, such as ordinary differential equations (ODEs) (Perelson, 1989; Baker et al., 1997; Chang et al., 2005; Vodovotz et al., 2006; Jarrett et al., 2015; Bonin et al., 2016), partial differential equations (PDEs) (Pettet et al., 1996; Su et al., 2009; Flegg et al., 2012; Pigozzo et al., 2013; Quintela et al., 2014), stochastic methods (Chao et al., 2004; Xavier et al., 2017), cellular automaton and agents (Celada and Seiden, 1992; Morpurgo et al., 1995; Kohler et al., 2000; Bernaschi and Castiglione, 2001; Pappalardo et al., 2018). On the other hand, very few papers were published describing the dynamics of SARS-CoV-2 (Almocera et al., 2020; Du and Yuan, 2020; Hernandez-Vargas and Velasco-Hernandez, 2020; Xavier et al., 2020), although some additional non-peer-reviewed papers can also be found on the internet. Three out four published works (Almocera et al., 2020; Du and Yuan, 2020; Hernandez-Vargas and Velasco-Hernandez, 2020) are based on the target cell-limited model proposed by Perelson (2002): a system of three ODEs to model target cells, infected cells, and viruses. In this work, we use a set of fifteen ODEs to model not only the virus but also immune cells, antibodies and cytokines.

Du and Yuan (2020) developed a mathematical model to investigate the dynamics of the immune response to influenza and the SARS-CoV-2 virus, including in their analysis the effects of a hypothetical antiviral drug on the SARS-Cov-2 infection. The model uses constants to represent the adaptive system CD8^+^ cells, IgM and IgG antibodies. The authors argue, based on their numerical results, that the innate immune system is the main responsible for clearing the influenza virus, while the adaptive system is the main responsible for controlling the SARS-CoV-2 virus. Their numerical results also suggest that the peak concentration of the adaptive immune cells for patients with COVID-19 is more likely to occur before the number of infected cells by SAR-CoV-2 reaches its peak (Du and Yuan, 2020). However, these results have not been validated: the viral loads found were not compared to experimental results.

A model similar to the one of Du and Yuan (2020) was proposed by Hernandez-Vargas and Velasco-Hernandez (2020), but including latent cells. The idea is that newly infected cells spend time in a latent phase, a concept similar to the “Eclipse phase” (Beauchemin et al., 2008). Another difference is that instead of using a constant to represent T cells (Du and Yuan, 2020), Hernandez-Vargas and Velasco-Hernandez (2020) used an equation to represent them. The viral loads obtained by numerical experiments were compared to values found in the literature (Wölfel et al., 2020), with good fitness between numerical and experimental results. The authors then present the Stability Analysis of their model (Almocera et al., 2020), which suggests that the SARS-CoV-2 virus replicates fast enough to overcome T cell response and cause infection.

Xavier et al. (2020) proposed a model based on five Ordinary Differential Equations to model the immune response to SARS-CoV-2. The model parameters and initial conditions were adjusted to cohort studies that collected viremia and antibody data. The results have shown that the model was able to reproduce both viremia and antibodies dynamics successfully. However, the model does not take into account the CRS. The mathematical model proposed in this paper can describe the immune response to SARS-CoV-2 for survivors and non-survivors. The difference between the two scenarios is the cytokine storm, which leads to a deregulated immune response in the second group.

A nonlinear differential equation model was proposed by Waito et al. (2016) to represent the dynamics of the CRS. The authors consider in their model that the rate of production of cytokines is dependent on interactions with other cytokines. The model was adjusted using type I interferon (IFN) receptor (IFNAR)-knockout mice data since mice lacking the IFNAR on their leukocytes experienced a profound cytokine storm (Waito et al., 2016). Although thirteen cytokines were considered in their model, the computational results have shown that TNF-*α*, IL-10, IL-6, and MIP-1*β*, have the largest effects on the dynamics of the cytokine storm. In this work, we do not distinguish the cytokines types nor consider the interactions among cytokines in their production rate.

The mathematical model proposed in this paper is an extension of a prior model of the adaptive immune response (Bonin et al., 2019). The original model (Bonin et al., 2019) had their parameters adjusted to reproduce the immune response against the Yellow Fever vaccine. The model of Bonin et al. (2019) considers the main cells and molecules present in the immune response against a virus such as antigen-presenting cells, CD4^+^ and CD8^+^ T cells, B cells and antibodies.

In order to represent the immune response to SARS-CoV-2, the model presented in this paper has slight differences from the model shown in our previous work (Bonin et al., 2019), and these differences are summarised in Table 1. Some changes were necessary to represent the hypothesis that some immune system cells can be infected by the SARS-CoV-2 virus (Davanzo et al., 2020; Grant et al., 2021). To implement these changes, a new population was included in the model (*I*, to represent immune defence cells infected by SARS-CoV-2) as well as new terms were included in the defence cells to represent their infection. Furthermore, the production of pro-inflammatory citokines was introduced in order to represent the dynamics of the CRS. To achieve this purpose, a new population was included (*C*, to represent the cytokine), as well as new terms were included to represent their production by the immune cells. Finally, we differentiate the production of antibodies into $I_{g}M$ and $I_{g}G$. These changes will be presented in the next section.

**TABLE 1:** Major differences between the models proposed in a previous work (Bonin et al., 2019) and in this work.

	Bonin et al. (2019)	This work
Number of equations	12	15
Number of parameters	32	38
Number of compartments	1	1
Number of considered populations	9	12

## 3 Materials and Methods

### 3.1 Mathematical Model

**FIGURE 1:**
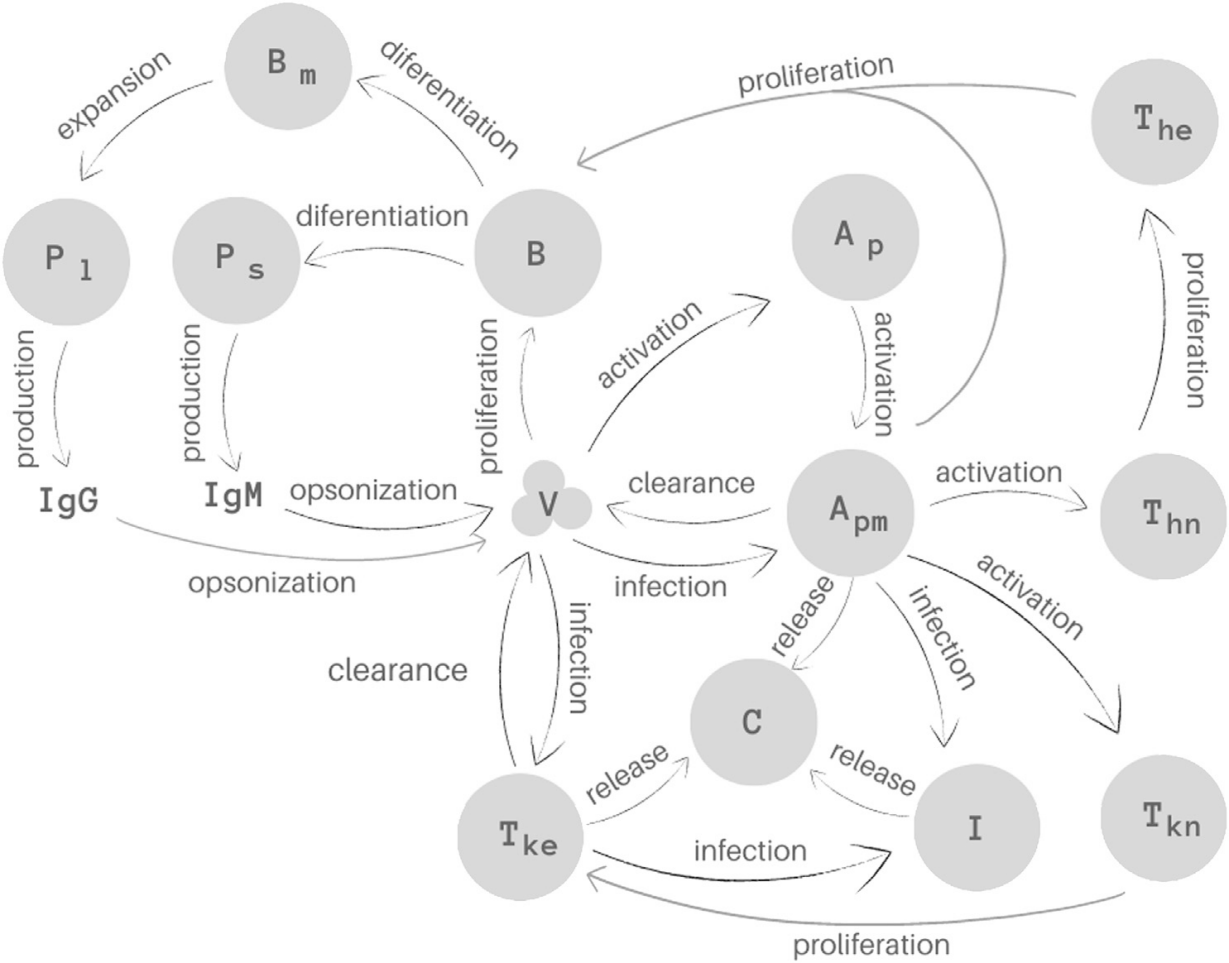
Representation of the 15 biological species and their interactions as considered in the mathematical model. Homeostasis is not illustrated in this Figure.

The model proposed in this work consists of a set of 15 Ordinary Differential Equations (ODEs), one to represent the behaviour of each population: virus (*V*), naive ($A_{p}$) and mature ($A_{pm}$) antigen-presenting cells (APCs), immune cells infected by the SARS-CoV-2 virus (*I*), naive ($T_{hn}$) and effector ($T_{he}$) T helper (CD4^+^) cells, naive ($T_{kn}$) and effector ($T_{ke}$) T Killer (CD8^+^) cells, B cells (*B*), short- ($P_{s}$) and long-lived ($P_{l}$) plasma cells, B memory cells ($B_{m}$), IgM ($Ig_{M}$) and IgG ($Ig_{G}$) antibodies and cytokines (*C*) (Figure 1).

The first equation describes the virus (V) behaviour. SARS-CoV-2 uses the cell surface receptor ACE2 to infect healthy cells (Catanzaro et al., 2020; Hoffmann et al., 2020; Shang et al., 2020), using its machinery to replicate itself. The replication is represented implicitly by the first term of Eq. 1, $\pi_{v}V$, where $\pi_{v}$ represents virus increase rate. The remaining terms of Eq. 1 represent the elimination of the virus by the immune system. The virus can be opsonized by antibodies, which facilitate its binding to receptor molecules present on the phagocytes (Paul, 2008). This is illustrated by the second and third term of Eq. 1. Effector CD8^+^ T-cells kills cells that are infected with viruses (Sompayrac, 2012). The term $k_{v1}VI_{gG}$ represents the elimination of virus due to the opsonization by $I_{gG}$ and the term $k_{v1}VI_{gM}$ represents the elimination of virus due to the opsonization by $I_{gM}$, where $k_{v1}$ is the opsonization rate. The term $k_{v2}VT_{ke}$ denotes specific viral clearance due to the induction of apoptosis of cells infected by the SARS-CoV-2 virus, where $k_{v2}$ is the clearance rate. Finally, $k_{v3}VA_{pm}$ denotes specific viral clearance due to phagocytosis by mature APCs, such as macrophages, where $k_{v3}$ is the clearance rate.

$$\frac{d}{dt}V=\pi_{v}V-k_{v1}VI_{gG}-k_{v1}VI_{gM}-k_{v2}VT_{ke}-k_{v3}VA_{pm}.$$ (1)

APCs are found in two stages, naive and mature (Murphy and Weaver, 2008). The second and third equations represent these two stages of the APCs, naive ($A_{p}$) and mature ($A_{pm}$). In this work, we consider that macrophages are the main APCs. In Eq. 2, the naive APCs homeostasis and activation are described by the first and second terms, respectively. The first term, $\alpha_{ap}\left(C+1\right)\left(A_{p0}-A_{p}\right)$, $\alpha_{ap}$ represents the homeostasis rate. Pro-inflammatory cytokines influence the homeostatic balance of the APCs (Murphy and Weaver, 2008). The term $\beta_{ap}A_{p}\frac{c_{ap1}V}{c_{ap2}+V}$ denotes the conversion of immature APCs into mature ones and, for this reason, the same term appears in Eq. 3 with positive sign. This function models growth combined with the saturation phenomenon (Goutelle et al., 2008).

$$\frac{d}{dt}A_{p}=\alpha_{ap}\left(C+1\right)\left(A_{p0}-A_{p}\right)-\beta_{ap}A_{p}\frac{c_{ap1}V}{c_{ap2}+V}.$$ (2)

In Eq. 3, which represents mature APCs, $\beta_{apm}A_{pm}V$ denotes $A_{pm}$ infection by the SARS-CoV-2 virus where $\beta_{apm}$ is the infection rate. The third term, $\delta_{apm}A_{pm}$, means the natural decay of the mature APCs, where $\delta_{apm}$ is the decay rate.

$$\frac{d}{dt}A_{pm}=\beta_{ap}A_{p}\frac{c_{ap1}V}{c_{ap2}+V}-\beta_{apm}A_{pm}V-\delta_{apm}A_{pm}.$$ (3)

The dynamics of the infected immune system cells is represented by Eq. 4. The first term, $\beta_{apm}A_{pm}V$, represents $A_{pm}$ infection and the second term, $\beta_{tke}T_{ke}V$, represents CD8^+^ T cells infection. The infection rates are, respectively, $\beta_{apm}$ and $\beta_{tk}$. Infected cells die with a rate $\delta_{apm}$.

$$\frac{d}{dt}I=\beta_{apm}A_{pm}V+\beta_{tke}T_{ke}V-\delta_{apm}I.$$ (4)

Equation 5 represents the population of naive CD4^+^ T cells ($T_{hn}$). The term $\alpha_{th}\left(T_{hn0}-T_{hn}\right)$ represents the homeostasis of CD4^+^ T cells, where $\alpha_{th}$ is the homeostasis rate. APCs are responsible for activating naive CD4^+^ T cells (Murphy and Weaver, 2008). The term $\beta_{th}A_{pm}T_{hn}$ denotes the activation of naive CD4^+^ T cells, where $\beta_{th}$ is the activation rate.

$$\frac{d}{dt}T_{hn}=\alpha_{th}\left(T_{hn0}-T_{hn}\right)-\beta_{th}A_{pm}T_{hn}.$$ (5)

Equation 6 represents effector CD4^+^ T cell population ($T_{he}$). The term $\pi_{th}A_{pm}T_{he}$ represents the proliferation of effector CD4^+^ T cells, where $\pi_{th}$ is the proliferation rate. The term $\delta_{th}T_{he}$ represents the natural death of these cells, with $\delta_{th}$ representing its death rate.

$$\frac{d}{dt}T_{he}=\beta_{th}A_{pm}T_{hn}+\pi_{th}A_{pm}T_{he}-\delta_{th}T_{he}.$$ (6)

Equations 7, 8 represent the population of naive ($T_{kn}$) and effector ($T_{ke}$) CD8^+^ T cells, respectively. In Eq. 7, the naive CD8^+^ T cells homeostasis and activation are described by the first and second terms, respectively. In the first term, $\alpha_{tk}\left(C+1\right)\left(T_{kn0}-T_{kn}\right)$, $\alpha_{tk}$ represents the homeostasis rate. The term $\beta_{tk}\left(C+1\right)A_{pm}T_{kn}$ denotes the activation of naive CD8^+^ T cells, where $\beta_{tk}$ is the activation rate. Pro-inflammatory cytokines have an influence on both the homeostatic balance and activation of naive CD8^+^ T cells.

$$\frac{d}{dt}T_{kn}=\alpha_{tk}\left(C+1\right)\left(T_{kn0}-T_{kn}\right)-\beta_{tk}\left(C+1\right)A_{pm}T_{kn}.$$ (7)

In Eq. 8, the term $\pi_{tk}A_{pm}T_{ke}$ represents the proliferation of effector CD8^+^ T cells. The terms $\beta_{tke}T_{ke}V$ and $\delta_{tk}T_{ke}$ represent the infection and death of effector CD8^+^ T cells, respectively.

$$\frac{d}{dt}T_{ke}=\beta_{tk}\left(C+1\right)A_{pm}T_{kn}+\pi_{tk}A_{pm}T_{ke}-\beta_{tke}T_{ke}V-\delta_{tk}T_{ke}.$$ (8)

Equation 9 represents both naive and effector B cells (*B*). These populations were not considered separately in order to simplify the model. The term $\alpha_{b}\left(B_{0}-B\right)$ represents the B cells homeostasis, where $\alpha_{b}$ is the homeostasis rate. The terms $\pi_{b1}VB$ and $\pi_{b2}T_{he}B$ represent the proliferation of B cells activated by the T-cell independent and T-cell dependent mechanisms (Sompayrac, 2012), respectively. The terms $\beta_{ps}A_{pm}B$, $\beta_{pl}T_{he}B$ and $\beta_{bm}T_{he}B$ denote the differentiation of active B cells into short-lived plasma cells, long-lived plasma cells and memory B cells, respectively. The activation rates are respectively given by $\beta_{ps}$, $\beta_{pl}$ and $\beta_{bm}$.

$$\begin{array}{cc} \frac{d}{dt}B=\alpha_{b}\left(B_{0}-B\right)+\pi_{b1}VB+\pi_{b2}T_{he}B-\beta_{ps}A_{pm}B & \\ -\beta_{pl}T_{he}B-\beta_{bm}T_{he}B. & \end{array}$$ (9)

Equation 10 represents the short-lived plasma cells ($P_{s}$) (Sompayrac, 2012). The term $\delta_{ps}P_{s}$ denotes the natural decay of short-lived plasma cells, where $\delta_{ps}$ is the decay rate.

$$\frac{d}{dt}P_{s}=\beta_{ps}A_{pm}B-\delta_{ps}P_{s}.$$ (10)

Equation 11 represents the long-lived plasma cells ($P_{l}$). The term $\delta_{pl}P_{l}$ denotes the natural decay of long-lived plasma cells, with $\delta_{pl}$ representing the decay rate. The term $\gamma_{bm}B_{m}$ represents the resupply of these cells by memory B cells, where $\gamma_{bm}$ is the production rate.

$$\frac{d}{dt}P_{l}=\beta_{pl}T_{he}B-\delta_{pl}P_{l}+\gamma_{bm}B_{m}.$$ (11)

Memory B cells ($B_{m}$) dynamics is represented by Eq. 12. The term $\pi_{bm1}B_{m}\left(1-\frac{B_{m}}{\pi_{bm2}}\right)$ represents the logistic growth of memory B cells, *i.e.*, there is a limit to this growth (Bonin et al., 2019). $\pi_{bm1}$ represents the growth rate, and $\pi_{bm2}$ limits the growth.

$$\frac{d}{dt}B_{m}=\beta_{bm}T_{he}B+\pi_{bm1}B_{m}\left(1-\frac{B_{m}}{\pi_{bm2}}\right)-\gamma_{bm}B_{m}.$$ (12)

Equations 13, 14 represents the production of antibodies. The terms $\pi_{ps}P_{s}$ and $\pi_{pl}P_{l}$ are the production of the antibodies by the short-lived and long-lived plasma cells, respectively. The production rates are given by $\pi_{ps}$ and $\pi_{pl}$, respectively. The terms $\delta_{am}I_{gM}$ and $\delta_{ag}I_{gG}$ denotes the natural decay of IgM and IgG antibodies, respectively, where $\delta_{am}$ and $\delta_{ag}$ are the decay rate.

$$\frac{d}{dt}I_{gM}=\pi_{ps}P_{s}-\delta_{am}I_{gM}.$$ (13)

$$\frac{d}{dt}I_{gG}=\pi_{pl}P_{l}-\delta_{ag}I_{gG}.$$ (14)

Finally, Eq. 15 describes the pro-inflammatory cytokine dynamics. In this equation, the first term, $\pi_{c_{apm}}A_{pm}$, represents the production of cytokines by $A_{pm}$, where $\pi_{c_{apm}}$ is the production rate. The second term represents the production of cytokines by infected cells, where $\pi_{ci}$ is the production rate. The third term represents the production of cytokines by $T_{ke}$ cells, where $\pi_{c_{tke}}$ is the production rate. Finally, the last term, $\delta_{c}C$, represents the cytokine natural decay, where $\delta_{c}$ is the decay rate.

$$\frac{d}{dt}C=\pi_{c_{apm}}A_{pm}+\pi_{ci}I+\pi_{c_{tke}}T_{ke}-\delta_{c}C.$$ (15)

We did not include in the model infected and non-infected epithelial cells because the model would be more complex, with more constants to adjust and, the worst, without data available to validate these cell populations along time. The use of implicit antigen replication does not affect the quality of the result, more specifically those related to the virus population, as our previous works have demonstrated (Bonin et al., 2018; Pigozzo et al., 2018; Bonin et al., 2019; Reis et al., 2019).

We are assuming that only mature cells are infected by the SARS-CoV-2 virus. In our simplification, we assume that the virus is located in the tissue, and that most of the naive cells are activated either in (or just after leaving) the bloodstream (APCs) or in the lymph nodes (CD4 and CD8) (Murphy and Weaver, 2008; Paul, 2008; Sompayrac, 2012). Another simplification adopted in this paper is that infected cells do not produce new virions: we assume that virus is mainly produced by the epithelial tissue despite the evidence that infected alveolar macrophages may support viral replication (Grant et al., 2021). We also assume that the phagocytic activity by infected cells does not occur. Finally, we assume that infected cells continue to produce pro-inflammatory cytokines and we implicitly consider the effects of different pro-inflammatory cytokines, the main effects of which are related to the IL-6 cytokine (Tanaka et al., 2014; Narazaki and Kishimoto, 2018; Zhang et al., 2020).

### 3.2 Experimental Data

Cohort studies available in the literature with people infected with SARS-CoV-2 were used to evaluate the mathematical model’s performance. In particular, viremia, antibodies (IgG and IgM), and cytokine levels for patients that survived and died due to COVID-19 were collected from three different papers.

The first paper presents a temporal profile of serial viral load from a set of 23 patients admitted at two hospitals in Hong Kong, all of them with laboratory-confirmed COVID-19 cases (To et al., 2020). Most of the viral load data reported in the paper were collected daily for 29°days from posterior oropharyngeal saliva samples. For this reason, we have used only this subset from this first paper. The number of patients who provided a sample on each day varies from one to ten. Data were extracted from the paper using the WebPlotDigitalizer tool (Rohatgi, 2020). WebPlotDigitizer is an on-line tool that can extract data in a semi-automatic way from graphics uploaded to the website.

The second paper presents antibody responses to SARS-CoV-2 (Long et al., 2020). A cohort composed of 285 Chinese patients confirmed to be infected with SARS-CoV-2 by RT-PCR assays were enrolled in this study from three hospitals. To measure the level of IgG and IgM against SARS-CoV-2, serum samples were collected at four different time intervals after the reported symptoms onset (Long et al., 2020). Antibody levels were measured using magnetic chemiluminescence, which provides values divided by the cutoff (S/CO) (Long et al., 2020), and were presented as $log_{2}\left(S/CO+1\right)$. The number of patients who provided a sample on each time interval varies from seven to one hundred and thirty. The dataset is available for download.

The third paper presents a retrospective cohort study with 191 adult inpatients from two Chinese hospitals (Zhou et al., 2020) diagnosed with COVID-19 according to WHO interim guidance and a clear outcome (dead or discharged) at the early stage of the outbreak. According to Zhou et al. (2020), 54 patients died during the stay in the hospital (non-survivors) while 137 were discharged (survivors). IL-6 plays a central role in the cytokine storm. For this reason, IL-6 data from this paper were collected using the WebPlotDigitalizer tool and used to adjust the cytokine levels of our mathematical model.

In all these three papers, the reported temporal profile of viremia, antibodies, and cytokines starts after symptom onset (Long et al., 2020; To et al., 2020; Zhou et al., 2020). This imposes an additional challenge because the exact day each patient has been infected is not clear. Epidemiological studies carried with 425 laboratory-confirmed COVID-19 cases in Wuhan, China, have estimated that the mean incubation period is about 5.2°days (Li Q. et al., 2020). Based on these results, we adjusted the cohort data accordingly to reflect the incubation period, *i.e.*, we have added 5°days at the beginning of each dataset to represent the time between the estimated infection until the symptom onset.

### 3.3 Parameter Estimation

One of the challenges related to the set of equations proposed to describe the immune response against the SARS-CoV-2 virus is the estimation of their parameters, i.e., find the values of the parameters which give the best fit to the set of the cohort studies. Unfortunately most of the parameters cannot be measured directly by experiments, and for this reason their biological ranges are unknown. Many distinct methods for non-linear systems can be used to estimate their values (Varah, 1982; Rodriguez-Fernandez et al., 2006; Schwaab et al., 2008). In this work we adopt the differential evolution (DE) optimisation method (Storn and Price, 1997).

Both initial conditions for the 15 variables and the 38 model parameters were calibrated to data obtained from cohort studies using DE. The DE was used to estimate each of the model parameters presented in Table 2, respecting the limits established for each one of them as presented in Tables 2 and 3.

**TABLE 2:** Bounds used for parameters calibration of the COVID-19 model for survivors. $V_{0}$ is the initial condition of the virus.

Parameter	Interval
$V_{0}$	[1.0, $1.0\times 10^{2}$]
$\pi_{v}$	[$8.0\times 10^{-1}$, $1.5\times 10^{0}$]
$k_{v1}$	[$1.0\times 10^{-5}$, $1.0\times 10^{-2}$]
$k_{v2}$	[$1.0\times 10^{-7}$, $1.0\times 10^{-4}$]
$k_{v3}$	[$1.0\times 10^{-4}$, $5.0\times 10^{-1}$]
$\beta_{ap}$	[$1.0\times 10^{-5}$, $1.0\times 10^{0}$]
$\beta_{apm}$	[$1.0\times 10^{-3}$, $1.0\times 10^{0}$]
$\beta_{tke}$	[$1.0\times 10^{-6}$, $1.0\times 10^{-4}$]
$\pi_{c_{apm}}$	[1.0, $5.0\times 10^{2}$]
$\pi_{ci}$	[$5.0\times 10^{-3}$, $1.0\times 10^{-1}$]
$\pi_{c_{tke}}$	[$1.0\times 10^{-5}$, $1.0\times 10^{-1}$]
$\delta_{c}$	[$1.0\times 10^{1}$, $1.0\times 10^{3}$]

**TABLE 3:** Bounds used for parameters calibration of the COVID-19 model for non-survivors.

Parameter	Interval
$\beta_{apm}$	[$1.0\times 10^{-3}$, $1.0\times 10^{0}$]
$\beta_{tke}$	[$1.0\times 10^{-6}$, $1.0\times 10^{-4}$]
$\pi_{ci}$	[$5.0\times 10^{-3}$, $1.0\times 10^{-1}$]

The idea used in the fitting is quite simple: minimise the difference between the model curves (viremia, IgG, IgM, and cytokines) to the given data (relative error) accordingly to Eq. 16.

$$\underset{p}{\text{min}}(\omega_{1}R_{E}(V,\hat{V})+\omega_{2}R_{E}(C,\hat{C})+\omega_{3}R_{E}(IgG,I\hat{g}G)+\omega_{4}R_{E}(IgM,I\hat{g}M)$$ (16)

where $\hat{V}\left(t\right)$ is the mean viral load, $I\hat{g}G\left(t\right)$ is the mean IgG antibody level, $I\hat{g}M\left(t\right)$ is the mean IgM antibody level, and $\hat{C}\left(t\right)$ represents the mean IL-6 level, *p* is the set of parameters to be estimated and $\omega_{n}$ is a weight. For this work, we used $\omega_{1}=\omega_{2}=1.0$ and $\omega_{3}=\omega_{4}=0.1$. The weights values were determined by the number of points in each dataset. For datasets with a small number of points, we used a small weight. This is due to the fact that, for small datasets, a small distance between the experimental data and the estimated value has a huge impact on error. $R_{E}$ represents the relative error between cohort data and the numerical result ($R_{E}\left(\lambda ,\hat{\lambda }\right)$), and is given by the two-norm of *λ* and $\hat{\lambda }$ divided by $\hat{\lambda }$.

The initial conditions for each variable are presented in Table 4. Table 5 presents the complete set of values used to calibrate our model to survivors’ data, including those found by the DE for the parameters listed in Table 2. The values that fit the model to non-survivors data are the same, except for those presented in Table 6, which were also obtained by the DE using the bounds presented in Table 3.

**TABLE 4:** Model variables and their initial values.

Variable	Description	Initial value	Unit
*V*	Virus	61	Copies/mL
$A_{p}$	Immature APCs	$10^{6}$	Cells/mL
$A_{pm}$	Mature APCs	0	Cells/mL
*I*	Infected cells	0	Cells/mL
$T_{hn}$	Naive CD4^+^ T cells	$10^{6}$	Cells/mL
$T_{he}$	Effector CD4^+^ T cells	0	Cells/mL
$T_{kn}$	Naive CD8^+^ T cells	$5\times 10^{5}$	Cells/mL
$T_{ke}$	Effector CD8^+^ T cells	0	Cells/mL
*B*	B Cells	$2.5\times 10^{5}$	Cells/mL
$P_{s}$	Short-lived plasma cells	0	Cells/mL
$P_{l}$	Long-lived plasma cells	0	Cells/mL
$B_{m}$	Memory B cells	0	Cells/mL
$I_{gM}$	Antibodies $I_{gM}$	0	S/CO
$I_{gG}$	Antibodies $I_{gG}$	0	S/CO
*C*	Cytokines	0	pg/mL

**TABLE 5:** Model parameters values that fit survivors’ data. The parameter presented in Table 2 were estimated using DE. The other values were based on the work of Bonin et al. (2019).

Parameter	Unit	Value
$\pi_{v}$	(day^-1^)	$1.47\times 10^{0}$
$k_{v1}$	(day^-1^ (mIU/ml)^-1^)	$9.82\times 10^{-3}$
$k_{v2}$	(day^-1^ (cells/mL)^-1^)	$6.10\times 10^{-5}$
$k_{v3}$	(day^-1^ (cells/mL)^-1^)	$6.45\times 10^{-2}$
$\alpha_{ap}$	(day^-1^(pg/mL)^-1^)	$1.0\times 10^{0}$
$\beta_{ap}$	(day^-1^(copies/mL)^-1^)	$1.79\times 10^{-1}$
$c_{ap1}$	(Copies/mL)	$8.0\times 10^{0}$
$c_{ap2}$	(Copies/mL)	$8.08\times 10^{6}$
$\delta_{apm}$	(day^-1^)	$4.0\times 10^{-2}$
$\beta_{apm}$	(day^-1^(copies/mL)^-1^)	$1.33\times 10^{-2}$
$\beta_{tke}$	(day^-1^(copies/mL)^-1^)	$3.5\times 10^{-6}$
$\alpha_{th}$	(day^-1^)	$2.17\times 10^{-4}$
$\beta_{th}$	(day^-1^ (cells/mL)^-1^)	$1.8\times 10^{-5}$
$\pi_{th}$	(day^-1^ (cells/mL)^-1^)	$1.0\times 10^{-8}$
$\delta_{th}$	(day^-1^)	$3.0\times 10^{-1}$
$\alpha_{tk}$	(day^-1^(pg/mL)^-1^)	$1.0\times 10^{0}$
$\beta_{tk}$	(day^-1^(pg/mL) ^-1^(cell/mL)^-1^)	$1.43\times 10^{-5}$
$\pi_{tk}$	(day^-1^(cells/mL)^-1^)	$1.0\times 10^{-8}$
$\delta_{tk}$	(day^-1^)	$3\times 10^{-1}$
$\alpha_{b}$	(day^-1^)	$3.58\times 10^{2}$
$\pi_{b1}$	(day^-1^(copies/mL)^-1^)	$8.98\times 10^{-5}$
$\pi_{b2}$	(day^-1^(cells/mL)^-1^)	$1.27\times 10^{-8}$
$\beta_{ps}$	(day ^-1^(cells/mL)^-1^)	$6.0\times 10^{-6}$
$\beta_{pl}$	(day^-1^(cells/mL)^-1^)	$5.0\times 10^{-6}$
$\beta_{bm}$	(day^-1^(cells/mL)^-1^)	$1.0\times 10^{-6}$
$\delta_{ps}$	(day^-1^)	$2.5\times 10^{0}$
$\delta_{pl}$	(day^-1^)	$3.5\times 10^{-1}$
$\gamma_{bm}$	(day^-1^)	$9.75\times 10^{-4}$
$\pi_{bm1}$	(day^-1^)	$1.0\times 10^{-5}$
$\pi_{bm2}$	(Cells/mL)	$2.5\times 10^{3}$
$\pi_{ps}$	(day^-1^(cells/mL)^-1^(S/CO))	$8.7\times 10^{-2}$
$\pi_{pl}$	(day^-1^(cells/mL)^-1^(S/CO))	$1.0\times 10^{-3}$
$\delta_{am}$	(day^-1^)	$7.0\times 10^{-2}$
$\delta_{ag}$	(day^-1^)	$7.0\times 10^{-2}$
$\pi_{c_{apm}}$	(day^-1^(cells/mL)^-1^(pg/ml))	$3.28\times 10^{2}$
$\pi_{ci}$	(day^-1^(cells/mL)^-1^(pg/ml))	$6.44\times 10^{-3}$
$\pi_{c_{tke}}$	(day^-1^(cells/mL)^-1^(pg/ml))	$1.78\times 10^{-2}$
$\delta_{c}$	(day^-1^)	$7.04\times 10^{2}$

**TABLE 6:** Model parameters values that fit non-survivors data. All other model parameters values were those from Table 5.

Parameter	Unit	Value
$\beta_{apm}$	(day^-1^(copies/mL)^-1^)	$1.51\times 10^{-2}$
$\beta_{tke}$	(day^-1^(copies/mL)^-1^)	$1.0\times 10^{-6}$
$\pi_{ci}$	(day^-1^(cells/mL)^-1^(pg/ml))	$9.96\times 10^{-2}$

### 3.4 Sensitivity Analysis

A sensitivity analysis (SA) was performed via main Sobol indices (Sobol, 2001). The SA is used to quantify the contribution of each uncertain model input $p_{i}$. Thus, Sobol indices support the process of identifying the parameters of the model that most affect the outputs, *Y*, predicted by the model. The main indices $S_{m}^{i}$ shows the portion of the total variance in *Y* that could be reduced if the exact value of $p_{i}$ is known, and it is computed as follows:

$$S_{m}^{i}=\frac{V\left[E\left[Y|p_{i}\right]\right]}{V\left[Y\right]},\text{ for }i\in \left[1,N\right],$$ (17)

where *N* is the number of parameters, V is the variance, and E the expected value. Therefore, a high value of $S_{m}^{i}$ indicates that the outputs of the models are more sensitive to $p_{i}$.

The sensitivity analysis was performed considering all parameters of the model. So, the main Sobol indices were evaluated considering all model parameter as uniform distributions, considering perturbations of 10% around the adjusted value, *i.e.* for a given model parameter value $v_{i}$ was built a uniform distribution ranging from [$0.9v_{i}$, $1.1v_{i}$].

### 3.5 Implementation

The model was implemented in the Python programming language. Numerical solution of the system of ODEs performed by the solve_ivp function, a member of the integrate package in the scipy library (Scipy, 2021). Among the integrate methods offered by this function it was used the Radau option, *i.e.* the fifth-order implicit Runge-Kutta method of the Radau IIA family. DE was implemented using the differential_evolution method available in the package optimize from the scipy. The SA was executed aided by SALib library (Herman and Usher, 2017).

## 4 Results

This section presents the predictions of the mathematical model presented in Section Materials and methods, comparing them with experimental data (Long et al., 2020; To et al., 2020; Zhou et al., 2020). Since one of the papers present cytokines levels for patients that survived and died due to COVID-19 (Zhou et al., 2020), we decided to divide our numerical simulations into two distinct scenarios: survivors and non-survivors.

This section also presents the results of the SA, identifying the ten parameters that most affect the outputs of the model.

### First Scenario: Survivors

**FIGURE 2:**
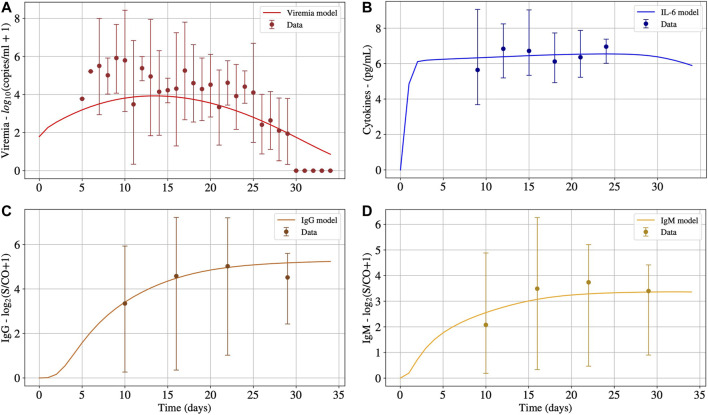
The numerical results presented in Panels A, B, C and D represent the solution of Eqs 1, 15, 14 and 13, respectively. These numerical solutions were fitted for patients without CRS. Interval used for initial conditions and parameters are given in Table 2.

Initially, as a validation step, we calibrated the model using data from patients that survived COVID-19 to check if the proposed model can fit the available cohort data. The model was able to represent viremia, cytokines, IgG and IgM from the patient data without CRS (Figure 2).

### Second Scenario: Non-survivors

**FIGURE 3:**
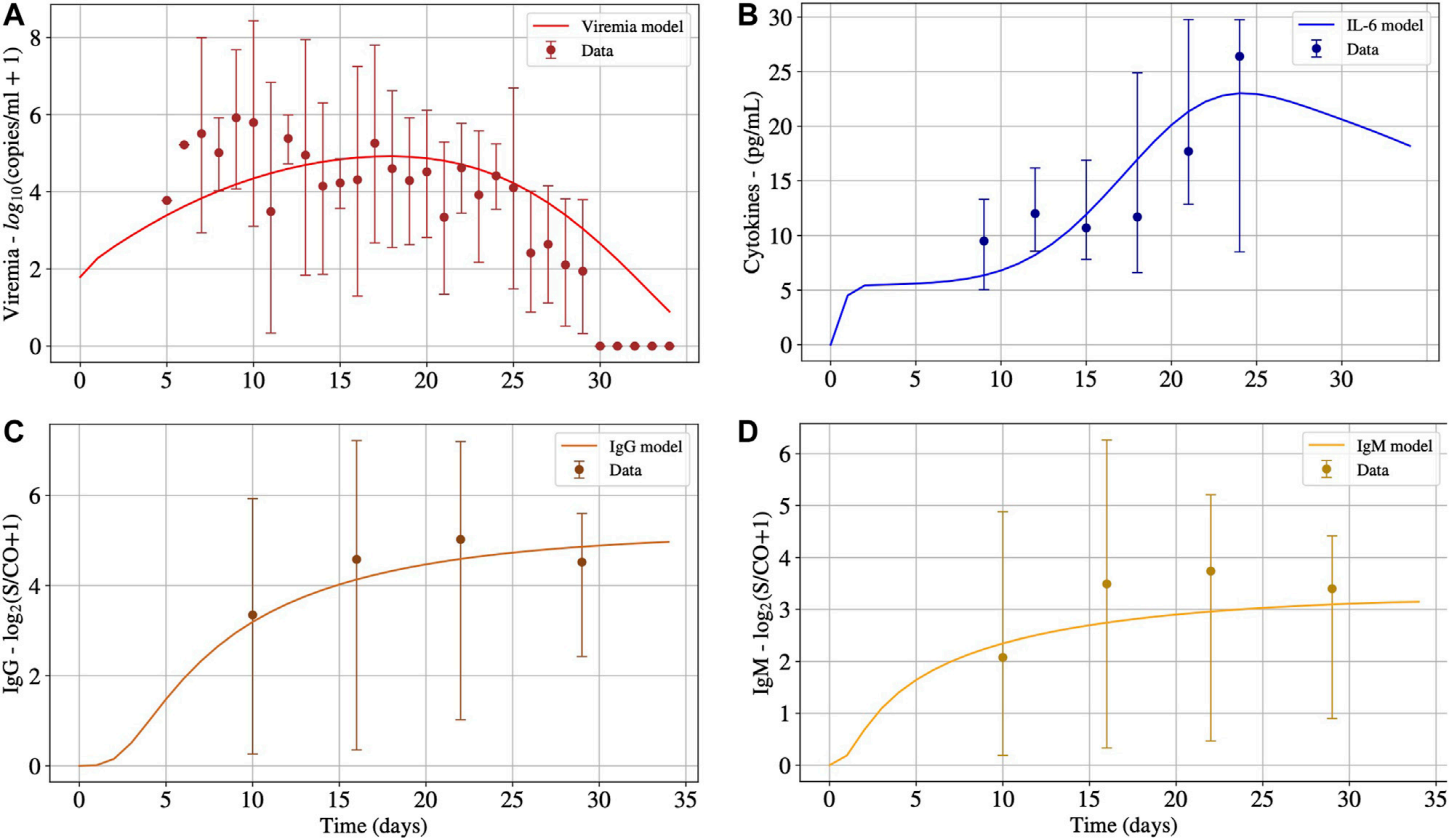
The numerical results presented in Panels A, B, C and D represent the solution of Eqs 1, 15, 14 and 13, respectively. These numerical solutions were fitted for patients with CRS adjusting the values of $\beta_{apm}$, $\beta_{tke}$ and $\pi_{ci}$, and keeping all other parameters and initial conditions values found in the previous adjust. Interval of the parameters are given in Table 3.

The second scenario simulates the immune response of non-survivors patients. Figure 3 presents the results. For this scenario, most of the parameters obtained in the first calibration were kept, except for $\pi_{ci}$, $\beta_{apm}$ and $\beta_{tke}$. We choose to modify only these three parameters because they are directly related to the hypothesis that SARS-CoV-2 infects effector APCs and T cells, causing the production of pro-inflammatory cytokines in a distinct rate of non-infected cells. More specifically, $\beta_{apm}$ and $\beta_{tke}$ represent the rate in which the SARS-CoV-2 infects effector APCs and CD8^+^ T cells, respectively, and $\pi_{ci}$ represents the rate in with infected immune cells produce pro-inflammatory cytokines. The new values for these three parameters were found after a new calibration using the DE optimisation method and are presented in Table 6.

**FIGURE 4:**
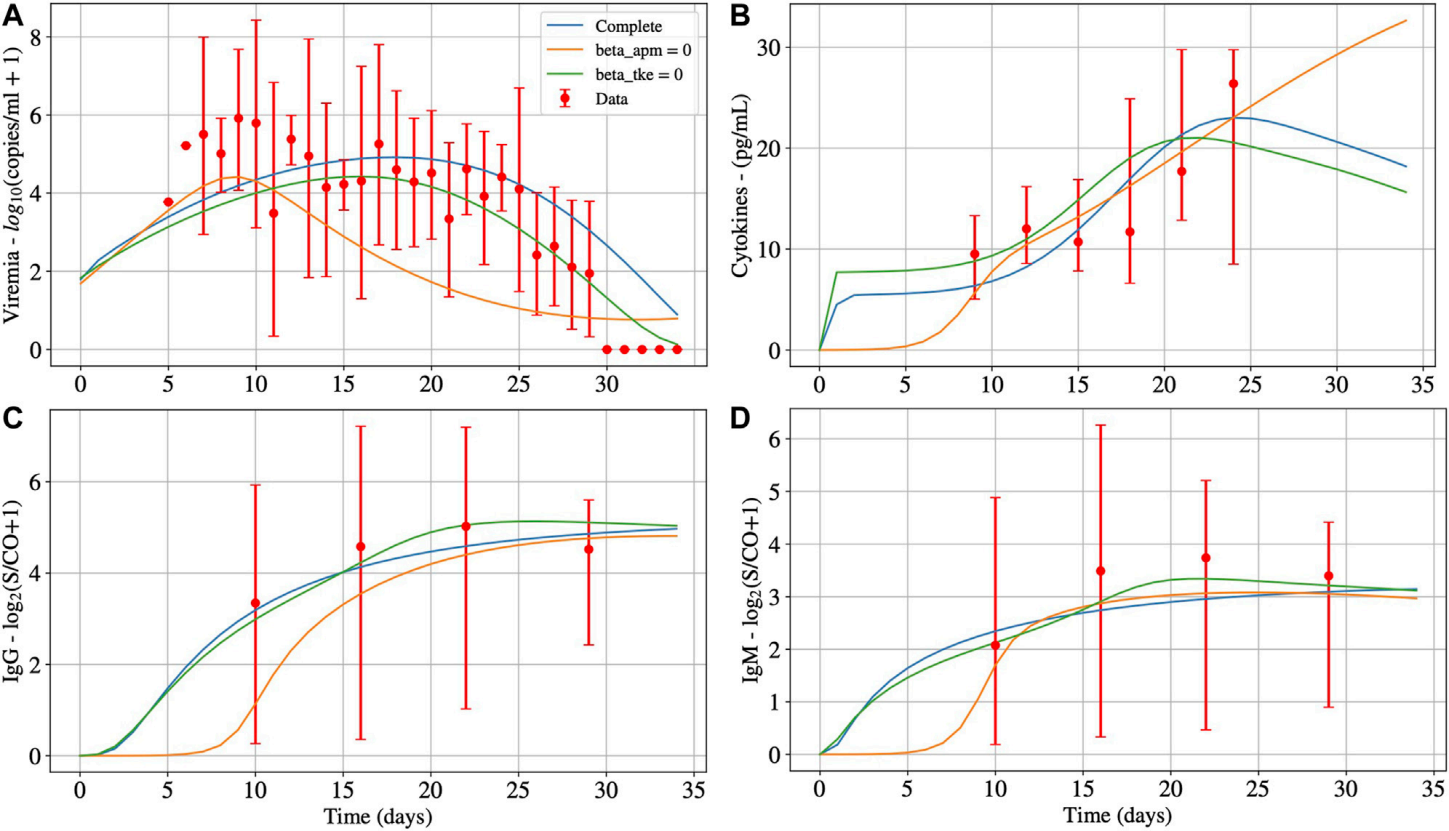
Deterministic numerical solution of Eqs 1, 15, 14 and 13 for the non-survivors case considering three hypothesis: 1) Infection of both effector APCs and CD8^+^ T cells (complete, with $\beta_{apm}\neq 0$ and $\beta_{tke}\neq 0$); 2) Infection of CD8^+^ T cells only ($\beta_{apm}=0$); and 3) Infection of effector APCs only ($\beta_{tke}=0$). Panels A, B, C and D present the results for viremia, cytokines, IgG and IgM, respectively.

Figure 4 presents the impacts of $\beta_{apm}$ and $\beta_{tke}$ in the cytokine levels. The idea is to evaluate each one separately: first, we consider that $\beta_{apm}$ is equal to zero, *i.e.*, the SARS-CoV-2 is not able to infect APCs. In this case, only CD8^+^ T cell can be infected and induce the production of large amounts of cytokines that can start the CRS. Then we do the opposite: we consider that $\beta_{tke}$ is equal to zero, *i.e.*, the SARS-CoV-2 is not able to infect CD8^+^ T cells. In this case, only APCs can be infected. Therefore, the model was re-fitted twice: in the first case, all model parameters were adjusted again, except by $\beta_{apm}$, which was set to zero. In the second case we did the same, but this time we considered that $\beta_{tke}$ is equal to zero. The results of both evaluations are then compared to the results obtained when both cells can be infected. Figure 4 presents the impacts of these changes for viremia, cytokines, IgG and IgM. Table 7 presents the new values of some model parameters to fit non-survivors data when $\beta_{apm}$ or $\beta_{tke}$ are equal to zero.

**TABLE 7:** Model parameters values that fit non-survivors’ data, when considering that only APCs ($\beta_{tke}=0$) or only T CD8^+^ cells ($\beta_{apm}=0$) are infected by SARS-CoV-2. The parameter presented in Table 2 were re-estimated using DE. All other model parameters values were those from Table 5.

Parameter	$\beta_{tke}=0$	$\beta_{apm}=0$
*V*	67	48
$\beta_{apm}$	$9.24\times 10^{-2}$	0.0
$\beta_{tke}$	0.0	$8.0\times 10^{-5}$
$\beta_{ap}$	$8.15\times 10^{-1}$	$1.52\times 10^{-4}$
$\pi_{c_{apm}}$	$3.67\times 10^{2}$	$4.81\times 10^{1}$
$\pi_{ci}$	$3.53\times 10^{-2}$	$2.03\times 10^{-2}$
$\pi_{c_{tke}}$	$1.55\times 10^{-2}$	$8.88\times 10^{-2}$
$\pi_{v}$	$9.70\times 10^{-1}$	$8.80\times 10^{-1}$
$k_{v1}$	$3.61\times 10^{-4}$	$3.04\times 10^{-3}$
$k_{v2}$	$8.00\times 10^{-5}$	$2.23\times 10^{-7}$
$k_{v3}$	$3.18\times 10^{-2}$	$1.03\times 10^{-1}$
δ_c	$4.15\times 10^{2}$	$8.25\times 10^{1}$

### Sensitivity Analysis

**FIGURE 5:**
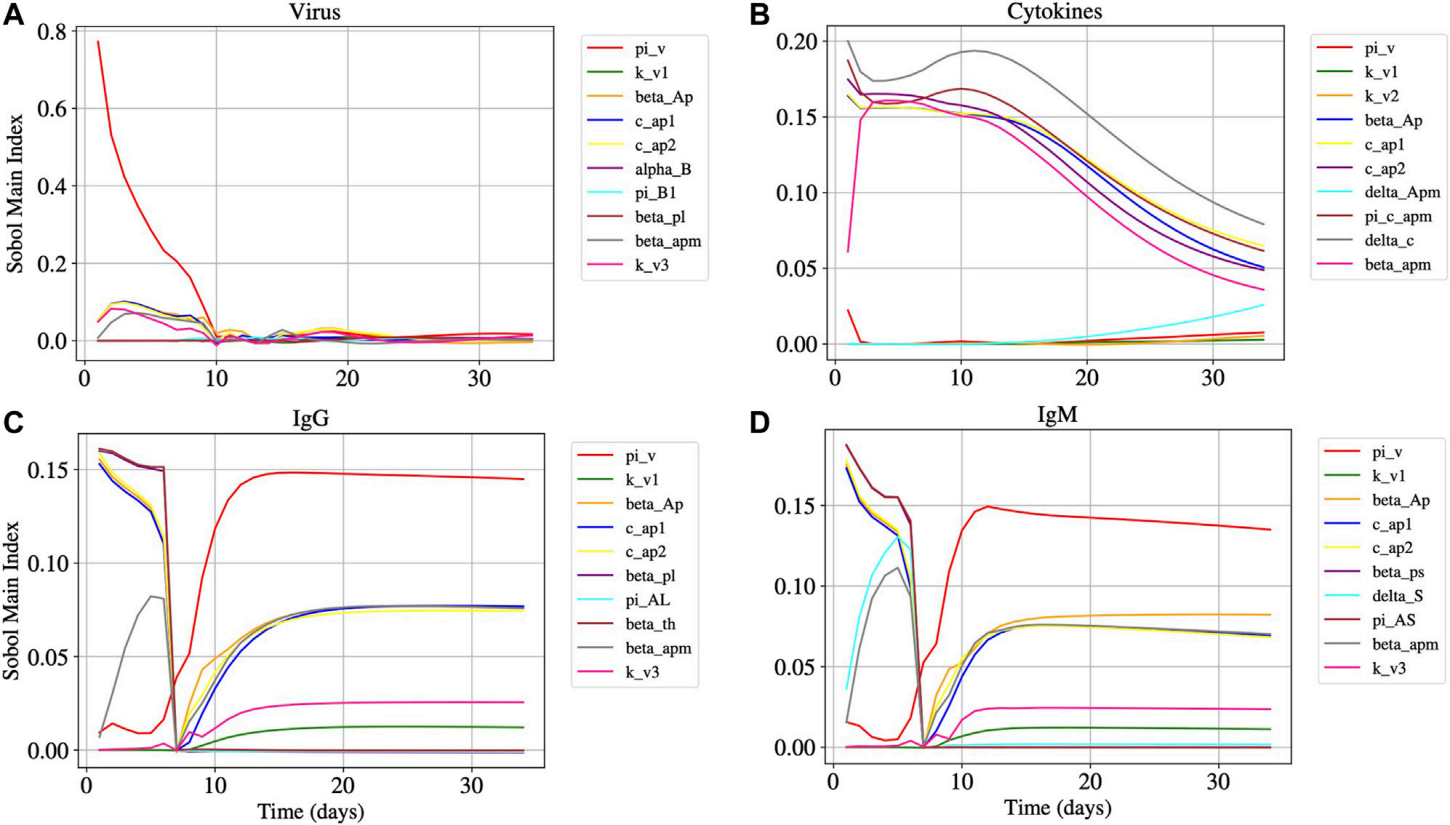
Panels A, B, C, and D represents the Sobol main index over time of Eqs 1, 15, 14 and 13, respectively. The main Sobol indexes were evaluated for all model parameters, but Panels A, B, C, and D presents the 10 parameters with largest influence in the results.

Figure 5 shows the main Sobol index ($S_{m}^{i}$) over time for Eqs 1, 13–15, considering that $\beta_{apm}\neq 0$ and $\beta_{tke}\neq 0$, *i.e.* both APCs and T CD8^+^ can be infected by SARS-CoV-2. Although the SA was performed for all 40 model parameters, some of then have small influence in the output produced for these equations and, for this reason, we decided to present in Figure 5 only the 10 parameters that have more impact in the results produced by the model.

## 5 Discussion

It can be seen in Panel A of Figure 2 that after 30°days, viruses were almost eliminated, but the concentrations of IL-6 (Panel B), IgG (Panel C), and IgM (Panel D) antibodies remain at high levels. Cytokines start to decrease after 27°days approximately. It is expected that IgG remains at high levels after the resolution of the inflammation. For how long these levels remain high is still an open question in the case of COVID-19. Moreover, after 30°days, the immune response has not ended, and the inflammation has not been regulated. In the presence of inflammation and viruses, the cells of the immune system continue to migrate, causing more inflammation. It is important to highlight that, after the complete elimination of the virus, the inflammation will decrease until it ceases, and the concentration of cells and molecules of the immune system will return to a homeostatic level. However, this process will occur slowly because the model does not consider an anti-inflammatory response.

As one can observe in Figure 3, after 30°days, the virus concentration (Panel A) tends to zero. On the other hand, the IL-6 concentration (Panel B) starts to increase considerably after ten days, achieving its peak around the $21^{st}$°day. After the peak, it decreases slowly. It can also be observed that the IL-6 concentrations are much higher in this scenario than in the previous one presented in Panel B of Figure 2, *i.e.*, the non-survivors peak is about three times higher than the survivors one. We believe that this huge increase in IL-6 levels was due to the CRS. Numerical results reveal that the CRS was caused mainly by effector APCs that are producing a considerable amount of pro-inflammatory cytokines. The infection of APCs was responsible for deregulating the innate immune response as well as for impairing the activation of the adaptive immune system. It is noteworthy that several other factors may contribute to CRS, among them a deficiency of the immune system in building an effective response against the virus at the beginning of the infection and a deficiency in controlling exacerbated inflammation.

In Figure 4, we observe that the infection of mature APCs, CD8^+^ T cells, or both cells simultaneously by SARS-CoV-2 can be determinant to the outcome of the disease. In other words, all hypotheses are plausible from a numerical perspective. The main differences in results are that the production of cytokines, IgG, and IgM (Panels B, C and D, respectively) starts later when $\beta_{apm}$ is equal to zero, *i.e.*, when only CD8^+^ T cells are infected by SARS-CoV-2. Also, infected CD8^+^ T cells impact both the day of the peak as well as its value (Panel A). We also observed that it is possible to obtain a similar result to the new fit of infected APC cells if we make $\beta_{apm}=0$ in the original adjustment, *i.e.*, those obtained when both mature APCs and CD8^+^ T cells are infected simultaneously by SARS-CoV-2. This result was not presented in this paper because it is close to the one obtained by the new fit, indicating that infected APCs influence the joint fitting more than infected CD8^+^ T cells.

From the results of the sensitivity analysis (Figure 5), we can observe that the most influential parameters in respect to the dynamics of the virus (Panel A), cytokines (Panel B), IgG (Panel C), and IgM (Panel D) populations are related to the APCs. We observe that $\beta_{ap}$, $c_{ap1}$ and $c_{ap2}$ are among the ten most influential parameters for the virus, cytokines, IgG and IgM populations. The parameter $\beta_{apm}$ is among the most influential for the virus, cytokines, IgG, and IgM populations. The virus replication rate $\pi_{v}$ has a great influence on the virus, IgG, and IgM dynamics.

It is worth mentioning that the experimental data presents a huge variation (observe that the scale adopted is log_10_), challenging the calibration process reported in Section 4. The severity of the disease could explain this. The literature reports that severe/critical patients tend to peak in the second week of illness, with values ranging from 5.57 to 9.66 log_10_ copies/mL, whereas mild/moderate patients tend to peak in the first week of illness, with values ranging from 3.25 to 6.40 log_10_ copies/mL (Lui et al., 2020). Thus, one possible explanation for the considerable variation observed in the viremia is that the dataset mixes patients with distinct disease severity degrees. In such a case, the numerical results represent an average value for distinct severity degrees. Nevertheless, some factors suggest a prevalence of severe/critical patients in the dataset: 1) the need for hospital admission; 2) the fact that the viremia peak observed in the numerical result occurs around the 15th°day, with a value about 6.0 log_10_ copies/mL, which is compatible with the one reported for severe/critical patients (Lui et al., 2020).

COVID-19 is a new disease, and for this reason, some studies and datasets seem to contradict each other. Different studies in the literature adopt distinct methods and metrics, so it is not easy to gather their data to create a much larger dataset. In addition, the experimental data used in this paper to calibrate the model comprises a reduced number of patients and measurements. In this sense, the results presented in this work have limitations due to the restrictions imposed by data availability.

This paper has analysed the hypothesis that the infection of immune defence cells causes the CRS in patients with COVID-19, mainly macrophages and CD8^+^ T cells, by the SARS-CoV-2. Although at first our numerical results suggest that this hypothesis may be correct, we must stress that the limitations described in this section could lead us to obtain an adjustment that supports, falsely, the hypothesis. Another limitation that can weaken our conclusions is that the combinations of values associated with other parameters except $\beta_{apm}$, $\beta_{tke}$, and $\pi_{ci}$, were not explored. We did not include parameters that are not related to the hypothesis evaluated in this paper and that can be associated to other theories found in the literature. For example, a paper from Garvin et al. (2020) indicates that the pathology of COVID-19 is likely the result of Bradykinin (BK) Storms rather than CRS. However, given the induction of IL-2 by BK, the two may be intricately linked. These theories could eventually lead to results similar or better to those obtained in our work.

Other techniques could be considered to distinguish survivors and non-survivors groups. The use of ODEs was motivated by our prior experience in using this tool to describe the dynamics of the immune response. Although we are not specialists in other techniques, such as deep learning, we believe that present limitations in the available data set can make it difficult or even impossible to use data driven models.

In the near future, we intend to explicitly represent the dynamics of different pro-inflammatory cytokines such as GM-CSF, TNF-*α*, IL-6, IL-8, among others. In addition, the model can be extended by considering anti-inflammatory responses through the incorporation of regulatory T cells (Treg cells), macrophages in a regulatory phenotype (Mreg phenotype), and anti-inflammatory cytokines such as IL-10. Finally, we also plan to use this model to reproduce the immune response against the SARS-CoV-2 vaccines.

## Data Availability

The original contributions presented in the study are included in the article/Supplementary Material, further inquiries can be directed to the corresponding author.
